# Peer-reviewed, high quality, worldwide information on all topics relevant to child and adolescent mental health

**DOI:** 10.1186/1753-2000-1-1

**Published:** 2007-06-26

**Authors:** Joerg M Fegert, Benedetto Vitiello

**Affiliations:** 1 University of Ulm, Ulm, Germany; 2 National Institute of Mental Health, Bethesda, USA

## Editorial

*Child and Adolescent Psychiatry and Mental Health *is a new, peer-reviewed, scientific journal devoted to the rapid dissemination of high-quality reports relevant to mental health of children and adolescents. It is the first open access electronic journal in child and adolescent mental health and aims to become an essential instrument of communication for researchers, clinicians, and policy makers.

The launch of this journal has been prompted by several considerations. First, mental health has acquired an increasingly prominent relevance to the health of our communities. After a century of major advances in general medicine and pediatrics, common birth complications and infectious diseases in the first years of life can now be successfully managed in economically developed countries. Consequently, behavioral and emotional disorders have become the primary causes of morbidity and mortality in childhood and adolescence [1]. Second, recent advances in basic and clinical neurosciences have documented that the most common psychiatric disorders in adulthood have their onset in the first two decades of life and can be best conceptualized as disturbances of "development". Third, while progress in the recognition and treatment of mental disorders in childhood and adolescence has been accomplished, the task of turning basic research findings into clinically useful applications still remains in front of us. Further progress will likely require sharing of knowledge across disciplines, such as psychiatry, psychology, pediatrics, genetics, neurosciences, public health, nursing, education, law, social work, social sciences, and economics. Thus, rapid dissemination of scientific information among multiple types of researchers and caregivers assumes an especially critical role. Hence, the creation of an internationally based, multidisciplinary journal focused on child and adolescent mental health whose access is open, without any barrier, to any interested reader with access to the Internet.

Empirical research has driven the progress in child psychiatry and clinical child psychology during the past decades. For further progress, the interface between basic science and clinical research appears especially important. More complex and sophisticated models of child-onset psychiatric disorders have been achieved by interdisciplinary collaborations between basic scientists and clinicians, such as the recent findings on specific gene-environment interactions. Another interface that has emerged as critically important is that between clinical research and implementation of empirically based interventions in clinical practice. To this end, it is essential to build collaborations between researchers and professionals involved in the care of children and adolescents, such as mental health specialists, pediatricians, social workers, and educators. This journal aims to help integrate basic science, clinical research and practical implementation of research findings.

In fact, even more than other medical disciplines, the field of child and adolescent psychiatry and mental health psychiatry is closely associated with various allied disciplines. Interdisciplinary collaboration, however, has been hindered by the compartmentalization of scientific publications and generally limited access to the print journals of neighboring disciplines. This journal aims to address these issues by taking a broad approach which inclusive of topics such a forensic psychiatry, prevention of psychiatric disorders, early interventions addressing the caregivers of infants, toddlers and preschoolers, mental health economics, and interventions specifically tailored to the needs of high-risk populations.

Because it can be accessed at no charge through the Internet, this journal is expected to attract a broader readership than traditional journals, including those from parts of the world where access to subscription journals is limited. In return, a broader and more diverse audience is likely to generate a greater variety of contributions. Because of the impact of social and cultural factors on the approach to diagnosis and treatment of mental illness, we believe that there will be a significant benefit from a wide exchange of information coming from different cultural approaches to mental health. In launching this new journal, we strongly feel encouraged by the *International Association for Child and Adolescent Psychiatry and Allied Professions (IACAPAP)*[2], an organization that is similarly devoted to promote the international collaboration of mental health professionals.

*Child and Adolescent Psychiatry and Mental Health *is committed to:

a) Publishing high quality scientific reports of new data that enhance knowledge on critical aspects of child and adolescent mental health, such as developmental psychopathology, etiopathogenesis, nosology, identification of risk factors, preventive interventions, treatment, bioethics, and economics implications.

b) Publishing challenging and thought provoking clinical case reports and commentaries on timely and controversial issues relevant to child and adolescent mental health.

c) Providing reviews of the literature on current and/or sometimes neglected issues of mental health of children and adolescents.

d) Fostering communication among disciplines relevant to child and adolescent mental health and thus contributing to interdisciplinary projects.

e) Rapid peer-review and publication process. The goal is to provide to the author(s) the reviewers' comments within six weeks from manuscript submission. Articles will be posted on the journal website immediately upon publication.

f) Open access of all publications to all interested readers through the Internet.

Open access, online publishing is a relatively new form of publication that has quickly spread out in the last few years especially in medicine and basic sciences. Many public and private research funding organizations support open access publishing or even require open access publishing in connection with their grants. This approach is driven by the idea that public money or money from scientific foundations should lead to scientific results that can be easily accessed by everybody without restrictions.

Because, by definition, open access journals do not levy subscription fees, the cost of publishing, maintaining and indexing articles has to be derived from other sources. For every article accepted for publication in the journal an article-processing charge is levied to cover the costs incurred by open access publication. Many universities, hospitals, and research institutions are members of BioMed Central [3], the journal's publisher. Authors from member institutions have the publication fee covered in part or in full by their institute. Authors from non-member institutes may be eligible for discounts and also have the opportunity to apply for a waiver (full details are available on the BioMed website [4])

Open access, online publishing has many advantages. Following a rigorous, but still expeditious, peer-review process, articles will be promptly posted by BMC on the Internet for anyone to download at no costs. All the articles published in C*hild and Adolescents Psychiatry and Mental Health *will immediately appear PubMed and become openly accessible through the Internet. Therefore, we encourage all potential contributors to consider fast publication in this new open access journal. In addition, authors retain the copyright of their article and have the right to reproduce and distribute as they please. We hope that readers all over the world will participate in the development of the journal and make it a success by informing their friends and co-workers about this new platform, and considering it for their next publication. We would like to thank our Associate Editors and the Editorial Office as well as the experts from BioMed Central for all their work that they have invested before we could launch this new journal. We feel strongly supported by a broad Editorial Board [5] with experts from many countries. Last but not least, we would like to thank all the reviewers who contribute to a fast evaluation of the manuscripts submitted to *Child and Adolescent Psychiatry and Mental Health*.
